# Factors associated with knowledge of obstetric danger signs and perceptions of the need for obstetric care among married men in northern Nigeria: a cross-sectional survey

**DOI:** 10.1186/s12884-019-2271-1

**Published:** 2019-04-11

**Authors:** Olugbenga Oguntunde, Jabulani Nyenwa, Farouk Musa Yusuf, Dauda Sulaiman Dauda, Abdulsamad Salihu, Irit Sinai

**Affiliations:** 1 UKAid/Nigeria MNCH2 Programme, No 17 Hospital Road, Nassarawa GRA, Kano, Nigeria; 2 Palladium, Abuja, Nigeria; 3 Palladium, London, UK; 4 Society for Family Health, Abuja, Nigeria; 5 Palladium, Washington, DC USA

**Keywords:** Maternal health, Male involvement, Social norms, Northern Nigeria

## Abstract

**Background:**

Male involvement in maternal, newborn and child health contributes to better health outcomes for women and their children, especially in restrictive societies. There is evidence that when men have better understanding of women’s health needs, attitudes toward utilization of maternal and child health services, of both women and men, are improved. Given the role of men as the ultimate decision makers in families in northern Nigerian society, this study assessed the determinants of men’s knowledge of danger signs in pregnancy and the continuum of obstetric care, and their perceptions of the importance of antenatal care utilization and health facility delivery.

**Methods:**

This was a cross-sectional descriptive study. Structured questionnaires with close ended questions were administered to 1627 married men who had at least one wife younger than 25 years in communities in Nigeria northern states of Kaduna and Katsina. We use crosstabulations and means to compare characteristics of study respondents in the two states, assessing statistical significance of the differences with χ^2^-square and Anova tests as appropriate, and logistic regressions to assess the determinants of knowledge and perceptions.

**Results:**

Knowledge of obstetric danger signs, especially during the postpartum period, was poor overall, but respondents were relatively more knowledgeable about danger signs during pregnancy and delivery compared with the postpartum period. Most perceived that antenatal care can reduce the risk of complications. Literate men were twice more likely to have positive health-behaviour perceptions. Wealth was positively associated with the perception that women should deliver in a health facility or hospital but did not have a statistically significant effect on the perception that antenatal care can reduce the risk of complications.

**Conclusions:**

While knowledge of obstetric danger signs was poor, literacy and household wealth significantly influenced knowledge of obstetric danger signs and perceptions that women should deliver at a health facility. Male involvement programmes need to ensure that men are empowered to understand obstetric danger signs along the continuum of obstetric care to improve perception and utilization of maternal health services for better maternal and newborn health outcomes.

**Electronic supplementary material:**

The online version of this article (10.1186/s12884-019-2271-1) contains supplementary material, which is available to authorized users.

## Background

Several countries in sub-Saharan Africa continue to carry a significant burden of maternal mortality, despite substantial progress worldwide in reducing the incidence of maternal deaths [1]. This is the case in northern Nigeria, a region characterized by health indicators that are among the poorest in the world. For example, available regional estimates for maternal mortality ratio in Nigeria stand at 1549 and 1026 per 100,000 live births for North-East and North-West regions respectively [2].

As in other African countries, women younger than 25 years in northern Nigeria are at particular risk for maternal morbidity and mortality [3, 4], yet adolescent marriage remains common in the region. According to the 2013 Demographic and Health Survey, the median age at first marriage of young women age 15–49 in northern Nigeria was 18.1, and many girls married when they were 12 years old or younger [5].

The literature demonstrates that in regions where adolescent marriages are common, restrictive social norms limit the mobility of married adolescents, and as a result, their access to, and utilization of, reproductive health and obstetric care is inadequate [6, 7]. In northern Nigeria, this one facet of the society negatively impacts utilization of maternal health services. The patriarchal nature of northern Nigerian society invests married men with social and economic powers that make them the leaders and ultimate decision makers within the family, community, and society [8, 9]. Explicit permission from husbands is required before married women of any age, but even more so the very young, can utilise maternal health services. Since women believe that this is as it should be [9], men in northern Nigeria often manage their wives’ fertility behaviours and their access to, and utilization of, available health care [10, 11].

Men in northern Nigeria often do not see the need to educate themselves on issues related to the health of their wives, because they see these as being ‘woman business’, even though women are not empowered to make health-behaviour decisions [12]. Women in the region are valued mainly for their reproductive functions and for the number of children they bear, yet their reproductive capacities are under the control of their husbands. They are highly dependent on their husbands, and defer to them in all household decisions, including those related to their own health [9, 13].

Studies from around the world, including in northern Nigeria, show that male involvement in utilization of maternal, newborn and child health plays an important role in better health outcomes of women, especially in restrictive societies. Improved attitudes, of both women and men, when men have better understanding of women’s health needs, have also been reported [14–17]. However, a recent systematic review, while documenting the overall usefulness of male involvement interventions in maternal and child healthcare outcomes, also noted evidence gaps on their impact on mortality and morbidity with recommendation on need for caution in their design and implementation to avoid reinforcing gender stereotypes that disempower women [18].

Given the role of men in northern Nigerian society as the ultimate decision makers in the family, and the documented importance of male involvement in maternal health, the Maternal Newborn and Child Health Programme (MNCH2), a five-year DFID UKaid-funded technical assistance project running from 2014 to 2019, implements a male support group intervention in six northern Nigeria states. The intervention aims at promoting male involvement in Maternal, Newborn and Child Health (MNCH) programming to increase women’s uptake and utilization of MNCH services. With focus on men who are married to young women less than 25 years, this study assessed the determinants of men’s knowledge of danger signs in pregnancy, delivery, and the postpartum period. It also evaluated their perceptions of the importance of attending antenatal care, and of delivering in a health facility.

## Methods

The study was undertaken in Kaduna and Katsina states, two of the six states where the Maternal Newborn and Child Health Programme (MNCH2) is currently active, both located in Nigeria’s northwest zone. It was a cross-sectional study and part of a broader operational research designed to assess men’s knowledge, attitudes, and perceptions related to various aspects of maternal, newborn and child health. Findings from this study were expected to influence the design and implementation of a male support group intervention aimed at improving utilization of MNCH services in MNCH2 programme states.

### Sample selection and fieldwork

To start, we selected one Local Government Area (LGA) from each of the three senatorial districts in each state and listed all communities in the selected LGAs. Several donor-funded maternal and child health programmes are active in Kaduna and Katsina. Since we did not want their activities to contaminate our data, we removed from the list communities supported by these programmes. We then randomly selected two communities from the list in each selected LGA, for a total of 12 study communities in the two states combined.

Using the formula *n*| = (*z*_1 − *α*_/*δ*)^2^ *p*(1 − *p*) for computation of sample size for descriptive studies with *p* = 50% at 95% confidence interval and precision level of 5%, we arrived at a sample size of 384. To adjust for non-response in some questions, we increased this figure to 420 per intervention community. We therefore interviewed 420 men each from these communities and in each of the two states, for a total of 840 per state, resulting in an overall sample size of 1680 in the two states combined.

To select respondents, we formed a cluster of about 200 neighbouring households in each selected community and used a random number generator on an Android phone to sample households for the interview. One man married to a woman younger than 25 was interviewed form each selected household (randomly selected if there was more than one eligible man in the household).

Fieldwork was undertaken in February 2017. Interviews were conducted by a team of 10 pairs of experienced and trained male research assistants, each pair led by a supervisor who determined respondents’ suitability for inclusion in the study and assured quality in data collection and overall study methodology. The study adapted and utilized the ‘Safe Motherhood questionnaire’ developed by JHPIEGO’s Maternal and Neonatal Health Programme [19](See Additional file 1). The adapted questionnaire was translated into the most commonly used local language (Hausa) and back translated to English to ensure accuracy and consistency and preloaded into android phones using Survey CTO application for direct data capturing. Interviews were conducted in Hausa in the privacy of respondents’ homes. The adapted questionnaire was pretested in a location not included in our study and further reviewed for accuracy.

### Analysis and variables

Data were imported from the Survey CTO into SPSS version 22 for analysis. We used cross-tabulations and means to compare characteristics of study respondents in the two states, assessing statistical significance of the differences with χ^2^-square and Anova tests as appropriate. We then used logistic regressions to assess the determinants of knowledge and perceptions. We examined five equations, using the following dependent variables. Details of the variables in the analysis, including their computation, are provided (see Additional file 2).

## Results

We interviewed a total of 1627 men in Kaduna and Katsina states combined, giving a response rate of 96.8%. As shown in Table 1, respondents in both states were mostly Muslim, with similar mean age. About a quarter of respondents in Katsina, and a third in Kaduna, were in polygamous marriages. Respondents in Kaduna were significantly more likely to be literate than those in Katsina. Less than 60% of respondents, in both states, had an electrical connection in their homes.

**Table 1:** Respondent profile

Characteristic	Kaduna *n* = 796	Katsina *n* = 831
Mean age	39.6	40.5
% Muslim	81.3	98.6
% in polygamous marriage	30.0	24.2
Mean number of children	4.9	4.4
% literate	73.1	55.2
% household connected to electricity	57.2	59.2
Mean household wealth index	31.0	26.1

We next assessed knowledge and perceptions about danger signs of pregnancy, delivery and the postpartum period as shown in Table 2. We observed that respondents in Kaduna were significantly more aware of danger signs in pregnancy, labour and delivery, than respondents in Katsina. Respondent from the two states were relatively more knowledgeable about danger signs during pregnancy with more than two thirds mentioning at least two danger signs. However, knowledge of danger signs during the first 2 days postpartum were relatively poorer with only about one third being able to mention at least two signs in Katsina. Also, significantly more Kaduna respondents said that at least one of their wives had experienced complications in pregnancy, labour, and the first two postpartum days. Almost all respondents in Kaduna and the majority in Katsina perceived that antenatal care can reduce the risk of complications, and significantly more respondents in Kaduna than Katsina said that women should deliver their babies in a health facility or hospital.

**Table 2:** Knowledge and experience of complications of pregnancy, labour and delivery

	Kaduna n = 796	Katsina n = 831
% mentioned at least two		
Danger signs in pregnancy	63.7***	42.4
Danger signs in labour and delivery	71.7***	62.1
Danger signs in first two postpartum days	53.9***	34.9
% Wife experienced at least one		
Pregnancy complication	52.5***	34.7
Labour complication	39.3***	32.4
First-two-days complication	44.2***	33.1
% said that antenatal care can reduce the risk of complications	94.3***	83.2
% said that women should deliver in health facility or hospital	66.3***	49.0
% said that a woman must have her husband’s permission to go to the health facility if		
She is pregnant	64.6***	57.9
She thinks she is in labour	54.4	54.3
She is delivering at home and there is a problem	48.9	51.3

*** denotes significance at the *p* < 0.01 level

To put it in context, we see that about half of respondents in both states said that when a woman goes into labour, as well as if she delivers at home and there is a problem, she needs to have her husband’s permission before going to a health facility. When we ran a cross-tabulation of the belief that women require their husband’s permission to go to the health facility when in labour or if she is delivering at home and there is a problem, against the perception that women should deliver in a health facility, we found no real differences between the groups – about half of men who perceived that women should deliver in a health facility also said that women should have their husband’s approval to go to the facility when in labour, and if she is delivering at home and there is a problem.

### Multivariate analysis

We start with examining the determinants of respondents’ awareness of danger signs in pregnancy, delivery, and the first two postpartum days. Results are shown in Table 3.

**Table 3:** Logistic regression of knowledge of danger signs during pregnancy, labour and delivery and first two days postpartum

	Odds Ratios					
	Mentioned at least two danger signs in pregnancy		Mentioned at least two danger signs in delivery		Mentioned at least two danger signs in the first two days postpartum	
	aOR (95%CI)	*P* value	aOR (95%CI)	*P* value	aOR (95%CI)	*P* value
Current age	1.000 (0.987–1.012)	0.962	1.008 (0.997–1.020)	0.148	1.002 (0.990–1.014)	0.720
In polygamous marriage	0.936 (0.677–1.294)	0.690	1.195(0.895–1.595)	0.228	1.153 (0.858–1.549)	0.346
Number of children	1.018 (0.981–1.056)	0.353	1.024(0.989–1.061)	0.177	1.010 (0.976–1.045)	0.569
Literate	1.095 (0.821–1.460)	0.536	1.175(0.914–1.511)	0.209	1.180 (0.901–1.546)	0.229
Electricity in household	1.387 (1.027–1.874)	0.033	1.105(0.850–1.436)	0.457	1.446 (1.098–1.904)	0.009
Wealth 2nd quartile	1.016 (0.712–1.449)	0.932	0.962(0.716–1.294)	0.800	0.800 (0.571–1.120)	0.193
Wealth 3rd quartile	1.292 (0.886–1.883)	0.183	1.881(1.349–2.623)	0.000	1.460 (1.035–2.059)	0.031
Wealth 4th quartile	1.555 (1.033–2.340)	0.034	2.560(1.766–3.711)	0.000	1.522 (1.052–2.203)	0.026
Wife experienced at least one complication in pregnancy	17.60 (17.59–13.399)	0.000				
Wife experienced at least one complication in labour			3.827(2.940–4.981)	0.000		
Wife experienced at least one complication in first two days postpartum					8.924 (6.960–11.443)	0.000
Location (Kaduna)	1.887(1.459–2.441)	0.000	1.285(1.021–1.618)	0.033	2.153 (1.696–2.733)	0.000
Constant	0.170	0.000	0.452	0.002	0.129	0.000
-2 log likelihood	1547.642		1844.496		1750.532	

Only relevant variables included in equation for each of the knowledge variables

Not surprisingly, having their wives experience complications had the most significant positive effect on all three knowledge variables. Men in Kaduna were twice more likely to know two or more danger signs in pregnancy and in the first 2 days postpartum than Katsina respondents. Age, marital status, number of children, and literacy levels did not have a statistically significant effect on knowledge. However, household wealth had a significant positive effect. For example, men in the 4th wealth quartile were two and a half times more likely to mention at least two danger signs in pregnancy, labour and delivery and first 2 days postpartum than men in the lowest wealth quartile.

We next examine the determinants of the perceptions that antenatal care can reduce the risk of complications, and that women should deliver in a health facility or a hospital. We used the same independent variables but add the relevant knowledge indicators as explanatory variables. Results are shown in Table 4.

**Table 4:** Logistic regression of perceptions of healthy behaviours

	Odds Ratios			
	Antenatal care can reduce the risk of complications		Women should deliver in health facility or hospital	
	aOR (95%CI)	*P* value	aOR (95%CI)	*P* value
Current age	1.012 (0.995–1.029)	0.169	1.006 (0.997–1.020)	0.252
In polygamous marriage	0.840 (0.543–1.299)	0.433	0.588 (0.895–1.595)	0.000
Number of children	1.016 (0.963–1.073)	0.558	0.981 (0.989–1.061)	0.239
Literate	2.204 (0.935–2.170)	0.000	2.027 (0.914–1.511)	0.000
Electricity in household	1.242 (1.027–1.874)	0.100	1.524 (0.850–1.436)	0.001
Wealth 2nd quartile	0.937 (0.618–1.421)	0.760	1.079 (0.716–1.294)	0.615
Wealth 3rd quartile	1.256 (0.731–2.158)	0.408	1.521 (1.349–2.623)	0.009
Wealth 4th quartile	1.651 (0.826–3.299)	0.156	2.428 (1.766–3.711)	0.000
Mentioned at least 2 pregnancy danger signs	1.670 (0.964–28.95)	0.067		
Mentioned at least 2 delivery danger signs	4.054 (2.737–6.006)	0.000	1.397 (2.940–4.981)	0.010
Mentioned at least 2 danger signs first two days postpartum	2.296 (1.223–4.307)	0.010	2.540 (1.883–3.426)	0.000
Wife experienced at least one complication in pregnancy	0.633 (0.356–1.126)	0.120		
Wife experienced at least one complication in labour	1.094 (0.589–2.030)	0.776	0.939 (0.684–1.288)	0.069
Wife experienced at least one complication in the first two days postpartum	1.451 (0.768–2.742)	0.252	0.767 (0.546–1.078)	0.127
Location (Kaduna)	2.455 (1.641–3.673)	0.000	1.686 (1.342–2.117)	0.000
Constant	0.608	0.162	0.235	0.000
-2 log likelihood	875.070		1890.266	

Only relevant variables included in equation for each of the ANC and health facility delivery variables

Our findings show that respondents in polygamous marriages were half as likely than those with only one wife to perceive that women should deliver in a health facility or hospital; literate men were twice more likely to have both of the positive health-behaviour perceptions. Wealth was positively associated with the perception that women should deliver in a health facility or hospital but did not have a statistically significant effect on the perception that antenatal care can reduce the risk of complications.

Of interest are the findings on the importance of knowledge of obstetric danger signs on perception about the perception that women should deliver their babies at a health facility. Respondents who recognized at least two danger signs in the first 2 days postpartum were two and a half times more likely than respondents who only recognized one danger sign or none, to perceive that antenatal care can reduce the risk of complications, as well as that women should deliver in a health facility or hospital.

## Discussion

This study examined knowledge and perceptions related to maternal health, of men who are married to young women in Kaduna and Katsina states in northern Nigeria. Our results show some differences between respondents in the two states, which reflect known demographic factors. Kaduna is not as homogenous as Katsina. There are more Christians living in the state, and the population is better educated [5]. These differences have significant implications for designing and implementing context specific MNCH intervention contents to ensure that relevant and location specific intervention are delivered to achieve maximal impact.

Our findings are consistent with results of studies from other parts of Africa which show an overall low awareness of maternal danger signs among men [18, 20–22]. While knowledge of maternal danger signs was clearly better in Kaduna, it was generally poor among most respondents. In addition, knowledge of danger signs during the postpartum period was particularly poor among most respondents. Poor knowledge of danger signs during the immediate postpartum period compared with other periods along the continuum of obstetric care has also been described elsewhere by similar studies [21, 23]. The poor knowledge of postpartum maternal danger signs compared with other periods along the continuum of obstetric care could probably be due to cultural factors that places more attention on pregnancy than the postpartum period. This has important programmatic implications such that male support group interventions to improve male involvement in maternal health ensure that participants to understand and appreciate danger signs along the whole continuum of obstetric care, especially the postpartum period.

Respondents in Kaduna showed better knowledge of danger signs along the continuum of pregnancy, labour and delivery, and the first two postpartum days. These differences are substantial, and were confirmed in the multivariate analysis, but we must consider how these variables are defined as we interpret these results. For this study, we coded the three knowledge variables as dichotomies, where 1 means that the respondent was aware of two or more danger signs, from a list of 10 items for pregnancy, seven for delivery, and 13 for the first 2 days postpartum; and 0 if they were aware of only one danger sign, or none at all. For example, when we say that 63.7% of men in Kaduna were aware of two or more danger signs in pregnancy, we also say that about a third of men knew only one of 10 danger signs, or not even one.

In both states, significantly more men perceived antenatal care as useful and that its utilization by pregnant women can reduce the risk of pregnancy, delivery and postpartum complications. However, fewer respondents perceived the need for women to deliver their babies in a hospital or other health facility. This also confirms the known literature from Nigeria and other parts of Africa about the importance that are attached to antenatal care attendance but that does not necessarily lead to facility delivery [18, 24].

The multivariate analysis of the perception that antenatal care can reduce the risk of complications, and that women should deliver in a health facility, confirms that knowledge of danger signs can result in more positive perceptions of health behaviour. It also appears that wealthier men have better perceptions of the need for obstetric care. Similarly, the multivariate analysis showed that household wealth was the only socio-economic factor to influence knowledge of danger signs in pregnancy, labour and delivery, and in the first 2 days postpartum.

## Study limitations

This study has some limitations. The study employed a cross-sectional design with limitations about cause and effect. This makes it impossible to establish a definitive causal relationship between our variables of interest. Some outcome variables for this study include men’s perception about benefit of ANC and health facility delivery for pregnant women and not actual spousal practices and may make attribution less strong. Nevertheless, our study came up with important findings that would be very useful in providing guidance about designing male support group intervention that could improve maternal and child health outcomes. In addition, the large number of men who participated in the study provided a reliable sample of our study population.

## Conclusions

This study revealed poor knowledge of obstetric danger signs among men in Kaduna and Katsina with significant differences between the two states. Literacy and household wealth significantly influenced knowledge of obstetric danger signs and the perception that women should deliver at a health facility. Male involvement programmes that aim to improve men’s knowledge of obstetric danger signs need to be context specific in designing and implementing interventions to address all continuum of obstetric care and the core determinants of knowledge determinants such as literacy and economic. Such programmes also need to ensure that interventions reach every strata of the community including the most economically disadvantaged men.

## Additional files

Additional file 1: Study questionnaire. (DOCX 203 kb)

Additional file 2: Variables and computation. (DOCX 16 kb)

## Abbreviations

ANC: Antenatal Care
LGA: Local Government Area
MNCH: Maternal, Newborn and Child Health
MNCH2: Maternal Newborn and Child Health Programme
