# Supplementary material for: Factors associated with knowledge of obstetric danger signs and perceptions of the need for obstetric care among married men in northern Nigeria: a cross-sectional survey
Source: BMC Pregnancy Childbirth. 2019 Apr 11;19:123. doi: 10.1186/s12884-019-2271-1 (PMC6458632; doi:10.1186/s12884-019-2271-1)
Supplement: Supplementary file 1 — Study questionnaire. (DOCX 203 kb) [file 12884_2019_2271_MOESM1_ESM.docx]

**Men’s Group Survey Questionnaire**

| A | INTERVIEWER'S NAME: |  |
| --- | --- | --- |
| B | STATE |  |
| C | LGA |  |
| D | COMMUNITY |  |
| E | TOWN/VILLAGE |  |
| F | NEIGHBOURHOOD |  |
| G | HOUSEHOLD NUMBER |  |
| H | NUMBER OF VISITS: | Visit 1 1  Visit 2 2  Visit 3 3 |

My name is........and I work for Akena Associates . You are invited to take part in a research study. Before you decide whether to participate, you need to understand why the research is being done and what it would involve. I will now tell you about the study. Please ask me if there is anything that is not clear, or if you would like more information. When all of your questions have been answered and you feel that you understand this study, I will ask you if you wish to participate in the study.

The MNCH2 project is undertaking this study about how a men mentor intervention among married men is influencing men’s knowledge about women’s and children’s health, and utilization of maternal, newborn and child health services. The study will enable MNCH2 programme to improve on how this intervention operates to ensure that it is effective.

As part of this study, we are interviewing married men in this community, who have a wife that is between 15 and 49 years old, and at least one child. You were randomly selected from among all married men living in this area who have a wife of reproductive age and at least one child. If you agree to take part in the study, you will participate in one interview, which will take no more than 30 minutes. The interview will include questions about maternal, newborn and child health and the men mentor programme, and how these can be improved.

I will not record your name or any other identifying information about you. This means that your answers could not be connected to you specifically from among all the participants. Your answers will not be shared with anyone. No one in your family or community will be able to know your answers. I will record your answers on my cell phone.

The interview will take place in a private room/area, where the conversation cannot be overheard. The study team will make every effort to protect your privacy and maintain the confidentiality of all the information that you provide.

The only risk associated with the survey is that others may know your responses. As I explained, we are taking steps to reduce this risk. There are no direct benefits to you for participating in the survey. However, the insights you provide may lead to better programmes that can improve the health of women and children.

Your participation in the study is completely voluntary. No one can make you participate if you do not wish to. If you agree to participate in the study, you may leave at any time without any consequences. If you decide to take part, you are free to decline to answer any questions.

Do you have any question? Do you wish to participate? Can we start the interview?

Ask:

May I begin the interview now:

Yes 1

No 2 (thank respondent and end interview)

**BACKGROUND CHARACTERISTICS**

| **No.** | **Question and Filters** | **Coding Category** | **Skip** |
| --- | --- | --- | --- |
| 101 | To start, please tell me, how old were you at your last birthday? | Age in completed years |  |
| 102 | What is the highest level of school you have attended? | Never attended school 1  Pre-primary/Nursery 2  Primary, not completed 3  Primary, completed 4  Secondary, not completed 5  Secondary, completed 6  College/University 7  Vocational/adult education 8  Islamiya 9  Other (specify) _______________ 99 |  |
| 103 | Please read this sentence to me  SHOW CARD TO RESPONDENT.  IF RESPONDENT CANNOT READ WHOLE SENTENCE,  PROBE:  Can you read any part of the sentence to me? | Cannot read at all 1  Able to read only parts of sentence 2  Able to read whole sentence 3  No card with required language ___________ 4  (specify language)  Blind/visually impaired 5 |  |
| 104 | What is your religion? | Islam 1  Christianity 2  Traditional 3  Other (Specify) ________________ 99 |  |
| 105 | Are you currently working, that is, doing something to generate income? | No 0  Yes 1 | 109 |
| 106 | If doing something to generate income, what do you do? | Professional/technical/managerial 1  Clerical 2  Sales/services (including trading) 3  Skilled manual 4  Unskilled manual 5  Agricultural 6  Other (specify _______________________ 99 |  |
| 107 | Do you usually work throughout the year, or do you work seasonally, or only once in a while? | Throughout the year 1  Seasonally 2  Once in a while 3 |  |
| 108 | Are you paid in cash or kind for this work or are you not paid at all? | In case only 1  Cash and kind 2  In kind only 3  Not paid 4 |  |
| 109m | Altogether, how many wives or live-in partners do you currently have? |  |  |
| 110m | How many wives or live-in partners have you ever had? |  |  |
| 111 | What was your age when you first got married? |  |  |
| 112 | Did you marry for the first time at about the age that men first get married in this community, or were you younger or older than typical age men first get married? | Typical 1  Younger 2  Older 3 |  |
| 114m | Thinking about your wife [wives], the one who was youngest when she first became pregnant. How old was she when she got pregnant for the first time? |  |  |
| 115m | How many children have you ever fathered in total? |  |  |
| 117m | How many of your live born children have died? |  |  |
| 118m | How many of your live born children are living with you? |  |  |
| 119m | How many of your live born children are living elsewhere? |  |  |
| 120 | How many children do you have who are 5 years old or younger? |  |  |

**KNOWLEDGE OF MATERNAL HEALTH**

**Read:** I will now ask you about the health of women and children: What you know about this and what is your own experience, starting with pregnancy.

| **No.** | **Question and Filters** | **Coding Category** | | | | **Skip** |
| --- | --- | --- | --- | --- | --- | --- |
| 201 | In your opinion, at what age should a woman have her first baby? | <15 1  15-19 2  20-24 3  25+ 4  When she starts her first period 5 | | | |  |
| 202 | I am going to read you some statements about pregnancy. Please tell me if you agree or disagree with each statement.   1. If a woman gets pregnant before 18 years of age, it may cause damage to her or her baby’s health 2. If a woman takes rest during pregnancy, then she would have difficulty during delivery 3. If a pregnant woman has some problem during pregnancy, only then she should consult a health provider, otherwise there is no need to consult them. 4. A pregnant woman should take a normal diet. There is no need to consume special food items during pregnancy 5. If the baby is delivered at home, he or she is healthier than if delivered in a health facility or a hospital 6. A pregnant woman should go to antenatal check-ups even if she is not sick | Agree | | Disagree | Don’t know/depends |  |
|  |  | 1  1  1  1  1  1 | | 2  2  2  2  2  2 | 8  8  8  8  8  8 |  |
| 203 | In your opinion, can unforeseen problems related to pregnancy occur during pregnancy, childbirth, and the first few days after childbirth, that can endanger the life of a woman? | No 0  Yes 1  Don’t know 98 | | | | 301  301 |
| 204 | What are some serious problems related to pregnancy that can occur while a woman is pregnant?  CIRCLE ALL MENTIONED  PROBE: Any other problems? | Bleeding A  Severe headache B  Blurred vision C  Convulsions D  Swollen hands/face E  High fever F  Loss of consciousness G  Difficulty breathing H  Severe weakness I  Severe abdominal pain J  Accelerated/reduced Fetal movement K  Water breaks without labor L  Other (specify) ____________________ X  Don’t know Z | | | |  |
| 205 | In your opinion, can a woman die from this [any of these] problems? | No 0  Yes 1  Don’t know 98 | | | |  |
| 206m | Did your wife [any of your wives] ever have any of these problems when she was pregnant? | No 0  Yes 1  Don’t know 98 | | | |  |
| 207 | What symptoms during pregnancy would indicate that there may be something going wrong with the pregnancy?  DO NOT READ OUT OPTIONS  SELECT ALL OPTIONS MENTIONED  PROBE: Any other problems? | Vaginal bleeding A  Pelvic or abdominal pain B  Persistent back pain C  Gush of fluid from the vagina D  Swelling of the hands/face E  Severe headaches/blurred vision F  Regular contractions earlier in pregnancy G  Baby does not move H  Other (specify) ______________________ X  Don’t know/None Z | | | | 209 |
| 208m | If your wife was to experience any of these symptoms during pregnancy, what first action would you take? | Take her to a health facility A  Buy medicine from pharmacy/shop B  Seek the help from TBA C  Seek the help of religious leader D  Seek the help of relatives E  Tell her to go to health facility F  Tell her to seek help G  Give her money to go seek help H  Other (specify) ______________________ X  Don’t know/None Z | | | |  |
| 209 | What are some serious problems that can occur during labour and childbirth?  DO NOT READ OUT OPTIONS  SELECT ALL OPTIONS MENTIONED  PROBE: Any other problems? | Severe bleeding A  Severe headache B  Convulsions C  High fever D  Loss of consciousness F  Labour lasting >12 hours G  Placenta not delivered soon after baby H  Other (specify) _______________________ X  Don’t know Z | | | |  |
| 210 | In your opinion, can a woman die from this [any of these] problems? | | No 0  Yes 1  Don’t know 98 | | |  |
| 211m | Did your wife [any of your wives] ever have any of these problems when she was giving birth? | | No 0  Yes 1  Don’t know 98 | | |  |
| 212 | What are some serious health problems that a woman can experience in the first two days after giving birth?  DO NOT READ OUT OPTIONS  SELECT ALL OPTIONS MENTIONED  PROBE: Any other problems? | | Severe bleeding A  Severe headache B  Blurred vision C  Convulsions D  Swollen hands/face E  High fever F  Vaginal secretions that smell bad G  Loss of consciousness H  Difficulty breathing I  Severe weakness J  Other (specify) ________________________ X  Don’t know Z | | |  |
| 213 | In your opinion, can a woman die from this [any of these] problems? | | No 0  Yes 1  Don’t know 98 | | |  |
| 214m | Did your wife [any of your wives] ever have any of these problems when she was giving birth? | | No 0  Yes 1  Don’t know 98 | | |  |
| 215 | What are some things that a woman can do to reduce the risk of problems in pregnancy and childbirth, and prepare for childbirth?  DO NOT READ OUT OPTIONS  SELECT ALL OPTIONS MENTIONED  PROBE: Any other things she can do? | | Receive antenatal care A  Take prenatal vitamins B  Identify mode of transport to health facility C  Save money D  Identify blood donor E  Other (specify) _______________________ X  Don’t know Z | | |  |
| 216m | Where do you think is the best place for your wife [wives] to deliver a baby? | | At home 1  At health facility or hospital 2  Either 3  Other (specify) _______________________ 99  Don’t know 98 | | | 218 |
| 217 | Why do you say that? | | Less or no cost 1  Safer 2  Better care 3  Near (or at) home 4  Convenient 5  Privacy 6  That’s what everyone does 7  Relatives say it’s best 8  Other (specify) _______________________ 99  Don’t know 98 | | |  |
| 218 | Has your child [any of your children] been delivered at home? | | No 0  Yes 1 | | | 220 |
| 219m | Think of when your child was born at home [your youngest child who was born at home] what was the main reason why the child was not delivered in a health facility or a hospital? | | Cost too much 1  Facility closed 2  Too far/no transportation 3  Don’t trust facility/poor quality service 4  No female provider 5  Child’s mother did not think it was necessary 6  I did not think it was necessary 7  Family did not think it was necessary 8  Other (specify) _______________________ 99  Don’t know 98 | | |  |
| 220 | Who should make the final decision on where a woman should give birth? | | Husband 1  The woman herself 2  Both husband and wife 3  Mother/mother-in-law 4  TBA 5  Other (specify) _______________________ 99  Don’t know 98 | | |  |
| 221 | Some women have problems during pregnancy and child birth. In your opinion, do women who are younger than 18 years are more likely or less likely than older women to have problems in pregnancy and child birth? | | More likdly 1  Less likely 2  I don’t know 98 | | |  |
| 222 | What about women who are older than 35? Are they more likely or less likely than younger women to have problems in pregnancy and child birth? | | More likdly 1  Less likely 2  I don’t know 98 | | |  |
| 223 | Are women who get pregnant less than two years after the birth of the last child more likely or less likely to have problems in their current pregnancy? | | More likdly 1  Less likely 2  I don’t know 98 | | |  |
| 224 | Are women who already have four or more children more likely or less likely to have problems in their next pregnancy? | | More likdly 1  Less likely 2  I don’t know 98 | | |  |
| 225 | What about women who are not educated? Are they more likely or less likely to have problems in pregnancy and child birth than women who had some education? | | More likdly 1  Less likely 2  I don’t know 98 | | |  |

**NEWBORN AND CHILD HEALTH**

| **No** | **Question and Filters** | **Coding Category** | | | | **Skip** |
| --- | --- | --- | --- | --- | --- | --- |
| 301 | Moving on to the health of newborn babies, what signs or symptoms would indicate that a baby who is less than a week old is sick and may be in danger?  DO NOT READ OUT OPTIONS  SELECT ALL OPTIONS MENTIONED  PROBE: any other signs or symptoms? | Difficult or fast breathing A  Yellow skin/eye colour (jaundice) B  Poor sucking or feeding C  Pus, bleeding, or discharge from  around the Umbilical cord D  Baby very small E  Skin lesions or blisters F  Convulsions or spasms G  Rigidity H  Baby is lethargic or unconscious I  Red or swollen eyes /pus in eyes J  Other (specify) ______________________ X  Don’t know Z | | | |  |
| 302 | In your opinion, can a baby die from this [any of these] problems? | No 0  Yes 1  Don’t know 98 | | | |  |
| 303 | Did your child [any of your children] ever have any of these problems in the first week after s/he was born? | No 0  Yes 1  Don’t know 98 | | | |  |
| 304 | What about children under the age of 5, what signs or symptoms would indicate that a child who is under the age of 5 is sick and may be in danger?  DO NOT READ OUT OPTIONS  SELECT ALL OPTIONS MENTIONED  PROBE: Any other problems? | Lethargy A  Severe weakness B  Changes in skin colour C  Vomiting D  Diarrhoea E  Dehydration F  Difficulty breathing F  Very fast breathing H  Fever I  Other (specify) ______________________ X  Don’t know Z | | | |  |
| 305 | In your opinion, can a child die from this [any of these] problems? | No 0  Yes 1  Don’t know 98 | | | |  |
| 306 | Did your child [any of your children] ever have any of these problems? | No 0  Yes 1  Don’t know 98 | | | |  |
| 307 | I am going to read you some statements about children’s health. Please tell me if you agree or disagree with each statement   1. If a child has a very high fever, s/he should be examined by a health provider 2. To remain healthy, young children should not be weighed very frequently 3. Diarrhoea in children is a minor problem and there is no need for mothers to be afraid of it 4. When a child develops diarrhoea, his intake of food and fluid should be restricted so that his intestines get rest 5. Children should always sleep under a bed net | Agree | | Disagree | Don’t know/depends |  |
|  |  | 1  1  1  1  1 | | 2  2  2  2  2 | 8  8  8  8  8 |  |
|  | If q113m = 0 (never fathered a child) skip to Q309 | | | | |  |
| 308 | If your child is very sick, who would you normally seek help from first? | Doctor/Nurse 1  CHW/TBA 2  Religious leader 3  Relatives 4  Other (specify) ___________________________ 99  Don’t know 98 | | | |  |
| 309 | Has your child [any of your children] ever received an immunisation? | No 0  Yes 1  Don’t know 98 |  | |  |  |
| 310m | I am going to read you some statements about Children’s health. Please tell me if you agree or disagree with each statement:   1. Immunisation can protect children against disease 2. At two months old a child is two young and should not receive any injection 3. Immunising children is against your religion 4. It is a father’s responsibility to make sure that his children are immunised on schedule | Agree | Disagree | | Don’t know/depends |  |
|  |  | 1  1  1  1 | 2  2  2  2 | | 8  8  8  8 |  |

**FAMILY PLANNING AND UNMET NEED**

QUESTIONS IN THIS SECITON PERTAIN ONLY TO HIS WIVES WHO ARE 15-45 YEARS OLD. IF HIS SENIOR WIFE IS OLDER, START WITH SECOND WIFE. IF SECOND ONE IS OLDER THAN 45, START WITH THIRD, ETC.

READ: Now I will ask you about ways that a couple can prevent or delay pregnancy.

| **No** | **Question and Filters** | **Coding Category** | | | **Skip** |
| --- | --- | --- | --- | --- | --- |
| 401 | Have you ever heard of ways or methods that a couple can use to delay or avoid pregnancy? | No 1  Yes 2 | | |  |
| 402 | Which methods have you heard of?  DO NOT READ OUT OPTIONS  SELECT ALL OPTIONS MENTIONED | Female sterilization A  Male sterilization B  Pill C  IUD D  Injectables E  Implants F  Condom G  Diaphragm/foam/jelly H  Standard Days Method I  Lactational Amenorrhea Method J  Periodic abstinence K  Withdrawal L  Herbal preparations (drink) M  Herbal preparations (douche) N  Spider web O  Beads/amulets P  Other traditional Q  Other (specify) ______________________ 99  Don’t know 98 | | |  |
| 403a | Is your senior wife (your wife, if man only has one wife of reproductive age) pregnant, or thinks she may be pregnant? | No 0  Yes 1  Don’t know 98 | | | 403b |
| 404a | Would you like your (senior) wife to have (a/another) child at any time in the future, or would you prefer that she never have any (more) children? | As God wills it 1  Have (a/another) child 2  No more/none 3  Undecided/don’t know 98 | | |  |
| 405a | Would you like your (senior) wife to become pregnant within the next year? | Yes 1  No 2  Says she can’t get pregnant 3  If God wills it 4  Don’t know 98 | | | 403b |
| 406a | Are you or your (senior) wife currently doing something or using any method to delay or avoid getting pregnant? | No 0  Yes 1  Don’t know 98 2 | | | 408a  408a |
| 407a | Which method are you and your (senior) wife using?  DO NOT READ OUT OPTIONS  SELECT ALL OPTIONS MENTIONED | Female sterilization A  Male sterilization B  Pill C  IUD D  Injectables E  Implants F  Condom G  Diaphragm/foam/jelly H  Standard Days Method I  Lactational Amenorrhea Method J  Periodic abstinence K  Withdrawal L  Herbal preparations (drink) M  Herbal preparations (douche) N  Spider web O  Beads/amulets P  Other traditional Q  Other (specify) ______________________ 99  Don’t know 98 | | |  |
| 408a | You have said that you do not want your (senior) wife to become pregnant in the next year, but you are not using any method to avoid pregnancy.  Can you tell me why you are not using a method?  Any other reason?  DO NOT READ OUT OPTIONS  SELECT ALL OPTIONS MENTIONED | PERCIVED RISK OF PREGNANCY  Not having sex AA  Infrequent sex AB  Menopausal/hysterectomy AC  Husband not fertile AD  Thinks she is sub-fecund or infecund AE  Postpartum amenorrheic AF  Breastfeeding AG  God’s will/up to God AH  OPPOSITION TO/DISAPPROVAL OF USE  Respondent opposed BA  Wife opposed BB  Wife won’t discuss it BC  Others opposed/fear of criticism/fear of  Losing status in family BD  Others will think she is a bad wife BE  Others will think he is weak BF  Religious prohibition BG  Believes it is immoral BH  Outsiders bring methods that harm  us/make us infertile BI  Want more children before using FP BJ  LACK OF KNOWLEDGE  Knows no Method CA  Don’t know where to obtain methods CB  Don’t know cost/thinks method is very  expensive CC  LACK OF ACCESS  Don’t have money to buy methods DA  No time to go to health facility DB  No way to get to health facility DC  Afraid someone will find out DD  METHOD-RELATED REASONS  Health concerns EA  Fear of side effects EB  Fear of infertility/sterility EC  Lack of access/too far ED  Costs too much EF  Inconvenient to use EG  Interferes with body’s normal processes EH  Method desired not available EI  Other (specify) ___________________________ X  Don’t know Z | | |  |
| 403b | Is your second wife pregnant, or thinks she may be pregnant? | No 0  Yes 1  Don’t know 98 | | | 403c |
| 404b | Would you like your second wife to have (a/another) child at any time in the future, or would you prefer that she never have any (more) children? | As God wills it 1  Have (a/another) child 2  No more/none 3  Undecided/don’t know 98 | | |  |
| 405b | Would you like your second wife to become pregnant within the next year? | Yes 1  No 2  Says she can’t get pregnant 3  If God wills it 4  Don’t know 98 | | | 403c |
| 406b | Are you or your second wife currently doing something or using any method to delay or avoid getting pregnant? | No 0  Yes 1  Don’t know 98 2 | | | 408b  408b |
| 407b | Which method are you and your second wife using?  DO NOT READ OUT OPTIONS  SELECT ALL OPTIONS MENTIONED | Female sterilization A  Male sterilization B  Pill C  IUD D  Injectables E  Implants F  Condom G  Diaphragm/foam/jelly H  Standard Days Method I  Lactational Amenorrhea Method J  Periodic abstinence K  Withdrawal L  Herbal preparations (drink) M  Herbal preparations (douche) N  Spider web O  Beads/amulets P  Other traditional Q  Other (specify) ______________________ 99  Don’t know 98 | | |  |
| 408b | You have said that you do not want your second wife to become pregnant in the next year, but you are not using any method to avoid pregnancy.  Can you tell me why you are not using a method?  Any other reason?  DO NOT READ OUT OPTIONS  SELECT ALL OPTIONS MENTIONED | PERCIVED RISK OF PREGNANCY  Not having sex AA  Infrequent sex AB  Menopausal/hysterectomy AC  Husband not fertile AD  Thinks she is sub-fecund or infecund AE  Postpartum amenorrheic AF  Breastfeeding AG  God’s will/up to God AH  OPPOSITION TO/DISAPPROVAL OF USE  Respondent opposed BA  Wife opposed BB  Wife won’t discuss it BC  Others opposed/fear of criticism/fear of  Losing status in family BD  Others will think she is a bad wife BE  Others will think he is weak BF  Religious prohibition BG  Believes it is immoral BH  Outsiders bring methods that harm  us/make us infertile BI  Want more children before using FP BJ  LACK OF KNOWLEDGE  Knows no Method CA  Don’t know where to obtain methods CB  Don’t know cost/thinks method is very  expensive CC  LACK OF ACCESS  Don’t have money to buy methods DA  No time to go to health facility DB  No way to get to health facility DC  Afraid someone will find out DD  METHOD-RELATED REASONS  Health concerns EA  Fear of side effects EB  Fear of infertility/sterility EC  Lack of access/too far ED  Costs too much EF  Inconvenient to use EG  Interferes with body’s normal processes EH  Method desired not available EI  Other (specify) ______________________ X  Don’t know Z | | |  |
| 403c | Is your third wife pregnant, or thinks she may be pregnant? | No 0  Yes 1  Don’t know 98 | | | 403d |
| 404c | Would you like your third) wife to have (a/another) child at any time in the future, or would you prefer that she never have any (more) children? | As God wills it 1  Have (a/another) child 2  No more/none 3  Undecided/don’t know 98 | | |  |
| 405c | Would you like your third wife to become pregnant within the next year? | Yes 1  No 2  Says she can’t get pregnant 3  If God wills it 4  Don’t know 98 | | | 403d |
| 406c | Are you or your third wife currently doing something or using any method to delay or avoid getting pregnant? | No 0  Yes 1  Don’t know 98 2 | | | 408c  408c |
| 407c | Which method are you and your third wife using?  DO NOT READ OUT OPTIONS  SELECT ALL OPTIONS MENTIONED | Female sterilization A  Male sterilization B  Pill C  IUD D  Injectables E  Implants F  Condom G  Diaphragm/foam/jelly H  Standard Days Method I  Lactational Amenorrhea Method J  Periodic abstinence K  Withdrawal L  Herbal preparations (drink) M  Herbal preparations (douche) N  Spider web O  Beads/amulets P  Other traditional Q  Other (specify) ___________________________ 99  Don’t know 98 | | |  |
| 408c | You have said that you do not want your third wife to become pregnant in the next year, but you are not using any method to avoid pregnancy.  Can you tell me why you are not using a method?  Any other reason?  DO NOT READ OUT OPTIONS  SELECT ALL OPTIONS MENTIONED | PERCIVED RISK OF PREGNANCY  Not having sex AA  Infrequent sex AB  Menopausal/hysterectomy AC  Husband not fertile AD  Thinks she is sub-fecund or infecund AE  Postpartum amenorrheic AF  Breastfeeding AG  God’s will/up to God AH  OPPOSITION TO/DISAPPROVAL OF USE  Respondent opposed BA  Wife opposed BB  Wife won’t discuss it BC  Others opposed/fear of criticism/fear of  Losing status in family BD  Others will think she is a bad wife BE  Others will think he is weak BF  Religious prohibition BG  Believes it is immoral BH  Outsiders bring methods that harm  us/make us infertile BI  Want more children before using FP BJ  LACK OF KNOWLEDGE  Knows no Method CA  Don’t know where to obtain methods CB  Don’t know cost/thinks method is very  expensive CC  LACK OF ACCESS  Don’t have money to buy methods DA  No time to go to health facility DB  No way to get to health facility DC  Afraid someone will find out DD  METHOD-RELATED REASONS  Health concerns EA  Fear of side effects EB  Fear of infertility/sterility EC  Lack of access/too far ED  Costs too much EF  Inconvenient to use EG  Interferes with body’s normal processes EH  Method desired not available EI  Other (specify) ___________________________ X  Don’t know Z | | |  |
| 403d | Is your fourth wife pregnant, or thinks she may be pregnant? | No 0  Yes 1  Don’t know 98 | | | 409 |
| 404d | Would you like your fourth wife to have (a/another) child at any time in the future, or would you prefer that she never have any (more) children? | As God wills it 1  Have (a/another) child 2  No more/none 3  Undecided/don’t know 98 | | |  |
| 405d | Would you like your fourth wife to become pregnant within the next year? | Yes 1  No 2  Says she can’t get pregnant 3  If God wills it 4  Don’t know 98 | | | 409 |
| 406d | Are you or your fourth wife currently doing something or using any method to delay or avoid getting pregnant? | No 0  Yes 1  Don’t know 98 2 | | | 408d  408d |
| 407d | Which method are you and your fourth wife using?  DO NOT READ OUT OPTIONS  SELECT ALL OPTIONS MENTIONED | Female sterilization A  Male sterilization B  Pill C  IUD D  Injectables E  Implants F  Condom G  Diaphragm/foam/jelly H  Standard Days Method I  Lactational Amenorrhea Method J  Periodic abstinence K  Withdrawal L  Herbal preparations (drink) M  Herbal preparations (douche) N  Spider web O  Beads/amulets P  Other traditional Q  Other (specify) ______________________ 99  Don’t know 98 | | |  |
| 408d | You have said that you do not want your fourth wife to become pregnant in the next year, but you are not using any method to avoid pregnancy.  Can you tell me why you are not using a method?  Any other reason?  DO NOT READ OUT OPTIONS  SELECT ALL OPTIONS MENTIONED | PERCIVED RISK OF PREGNANCY  Not having sex AA  Infrequent sex AB  Menopausal/hysterectomy AC  Husband not fertile AD  Thinks she is sub-fecund or infecund AE  Postpartum amenorrheic AF  Breastfeeding AG  God’s will/up to God AH  OPPOSITION TO/DISAPPROVAL OF USE  Respondent opposed BA  Wife opposed BB  Wife won’t discuss it BC  Others opposed/fear of criticism/fear of  Losing status in family BD  Others will think she is a bad wife BE  Others will think he is week BF  Religious prohibition BG  Believes it is immoral BH  Outsiders bring methods that harm  us/make us infertile BI  Want more children before using FP BJ  LACK OF KNOWLEDGE  Knows no Method CA  Don’t know where to obtain methods CB  Don’t know cost/thinks method is very  expensive CC  LACK OF ACCESS  Don’t have money to buy methods DA  No time to go to health facility DB  No way to get to health facility DC  Afraid someone will find out DD  METHOD-RELATED REASONS  Health concerns EA  Fear of side effects EB  Fear of infertility/sterility EC  Lack of access/too far ED  Costs too much EF  Inconvenient to use EG  Interferes with body’s normal processes EH  Method desired not available EI  Other (specify) ____________________ X  Don’t know Z | | |  |
| 409m | Have you (or your wives) ever done or used any method to delay or avoid getting pregnant in the past? | No 0  Yes 1 | | |  |
| 410 | Which method have you used in the past?  DO NOT READ OUT OPTIONS  SELECT ALL OPTIONS MENTIONED | Female sterilization A  Male sterilization B  Pill C  IUD D  Injectables E  Implants F  Condom G  Diaphragm/foam/jelly H  CycleBeads I  Lactational Amenorrhea Method J  Periodic abstinence K  Withdrawal L  Herbal preparations (drink) M  Herbal preparations (douche) N  Spider web O  Beads/amulets P  Other (specify) ____________________ X  Don’t know Z | | |  |
| 411 | Who should decide whether or not to use family planning? | The husband 1  The wife 2  Both husband and wife 3  Mother/mother-in-law 4  Other (specify) _________________ 98 | | |  |
| 412m | In the past 6 months, have you discussed family planning with your wife [any of your wives]? | No 0  Yes 1 | | |  |
| 413 | I am going to read you some statements about the use of methods for preventing or delaying pregnancy. Please tell me if you agree or disagree with each statement:   1. It is good to have many children because they can help with household tasks 2. Women who use family planning are straying from the correct path or are immoral 3. Women who use family planning look better than women who do not use family planning 4. The family planning methods provided by the health programs in this community are difficult to use 5. Couples who practice family planning and have fewer children are better able to provide for their family 6. Using family planning is bad for a woman’s health 7. Only God can decide the number and timing of children a couple has 8. Family planning is something that people from outside our community want us to do for their benefit, not ours | Agree | Disagree | Don’t know/depends |  |
|  |  | 1  1  1  1  1  1  1  1 | 2  2  2  2  2  2  2  2 | 8  8  8  8  8  8  8  8 |  |

**MATERNAL AND CHILD HEALTH SERVICES**

| **No** | **Question and Filters** | **Coding Category** | | | | | | **Skip** |
| --- | --- | --- | --- | --- | --- | --- | --- | --- |
| 501 | Next I will ask you about health services that are available to you in this community. Please think about the health facility nearest to where you live. I am going to read you some statements about that health facility. Please tell me if you agree or disagree with each statement   1. The facility is not far from my home 2. The facility has adequate qualified staff 3. The facility does not have all the medicines that my family and I need 4. It is safe for women to give birth in the facility 5. Services in the facility are too expensive 6. Health providers in the facility are usually polite | Agree | Disagree | | Don’t know/depends | | |  |
|  |  | 1  1  1  1  1  1 | 2  2  2  2  2  2 | | 8  8  8  8  8  8 | | |  |
| 502 | Where or who do you think women in this community should go to receive health care when pregnant? | Government health facility 1  Private health facility 2  Private clinic 3  Chemist 4  TBA 5  Prayer homes 6  No one 7  Other (specify) _____________________ 99 | | | | | |  |
| 503 | Where do you think women in this community should go for child birth? | Government health facility 1  Private health facility 2  Private clinic 3  Chemist 4  TBA 5  Prayer homes 6  Stay at home 7  Home of relatives 8  Home of friends 9  Other (specify) _____________________ 99 | | | | | |  |
| 504 | I will now mention several situations in which a woman may need to or want to go to a health facility. For each situation please tell me if the woman must have her husband’s permission before going to the facility or not. Mush she have her husband’s permission to go to the health facility if:   1. she is pregnant? 2. she thinks childbirth is starting? 3. she is delivering the baby at home and there is a problem? 4. she is sick or is not feeling well? 5. A child is sick or is not feeling well? 6. A child should be immunised? | No | | | | Yes | |  |
|  |  | 0  0  0  0  0  0 | | | | 1  1  1  1  1  1 | |  |
| 505 | Think about health facilities in this area. What maternal health services are provided there?  SELECT ALL OPTIONS MENTIONED, THEN PROBE FOR OPTIONS NOT MENTIONED AND CIRCLE IF RESPONDENTS THINK SERVICE IS PROVIDED | ANC 1  Family planning 2  Treatment of women who are sick 3  Treatment of sexually transmitted infections 4  Prevention of mother to child transmission  of HIV 5  Cervical cancer screening 6  Delivery services 7  Postnatal services 8  Other (specify) ____________________ 88  Don’t know 99 | | | | | |  |
| 507 | I am going to read you some statements about quality of care in health facilities in this community. Please tell me if you agree or disagree with each statement  Health providers in the facility are usually friendly and polite  Health providers in the facility are often rushed  Health providers usually provide women with the information they need  Facilities in this community do not have enough equipment  Health providers in facilities in this community care about women’s health  Health facilities usually have the needed drugs and medicines  Delivering a baby in a health facility is expensive  Often there is no privacy during consultations in health facilities  Health providers in the facilities are well trained  Health facilities cannot manage complications | Agree | | Disagree | | | Don’t know/depends |  |
|  |  |  | |  | | |  |  |
| 508m | The last time that your wife (any of your wives) was pregnant, did she attend ANC services? | No 0  Yes 1  Don’t know 99 | | | | | | 513  513 |
| 509 | Where did she go for ANC? | General hospital 1  Private hospital 2  Primary health care facility 3  Private clinic 4  Prayer home 5  TBA home 6  Our home 7  Other (specify) ____________________ 99 | | | | | |  |
| 512 | Who paid for the ANC services | Service was free 1  I paid 2  Wife paid 3  A relative paid 4  Insurance paid 5  The community paid 6  Other (specify) ______________________ 99 | | | | | |  |
| 513 | What are some reasons that women in this community do not attend ANC during pregnancy? | It is not necessary 1  They do not know where to go for service 2  ANC is expensive 3  Facility is too far 4  Their husbands do not let them 5  They do not want service from male provider 6  Service is not good 7  Women are busy and have no time for ANC 8 | | | | | |  |
| 504 | What are the reasons that women in this community deliver their babies at home? | Childbirth is safer at home 1  It is customary to deliver at home 2  Facility delivery is expensive 3  Facility is too far 4  Their husbands do not let them 5  They do not want service from male provider 6  Service is not good 7 | | | | | |  |

**PARTNER COMMUNICATION**

| **No.** | **Question and Filters** | **Coding Category** | **Skip** |
| --- | --- | --- | --- |
| 601m | In your household, who usually makes decision about major household purchases: you, your wife (wives), you and your wife (wives) jointly, or someone else?? | Respondent 1  Wife (wives) 2  Respondent and wife (wives) jointly 3  Someone else 4  Other (specify) _____________________ 99 |  |
| 602m | Who usually makes decisions about healthcare for your wife (wives): you, your wife (wives), you and your wife (wives) jointly, or someone else? | Respondent 1  Wife (wives) 2  Respondent and wife (wives) jointly 3  Someone else 4  Other (specify) _____________________ 99 |  |
| 603m | Who usually makes decisions about healthcare for your children: ): you, your wife (wives), you and your wife (wives) jointly, or someone else? | Respondent 1  Wife (wives) 2  Respondent and wife (wives) jointly 3  Someone else 4  Other (specify) _____________________ 99 |  |
| 604m | Would you say that the decision to use a method to delay or avoid pregnancy is mainly your decision, your wife (wives)’s decision, or a joint decision? | Husband 1  Wife 2  Joint decision 3  Other (specify) ____________________ 99 |  |
| 605m | In the past six months, how often did you and your wife (youngest wife) talk about planning your family, and whether or not to use a method to delay or avoid pregnancy? | Never 1  Once or twice 2  More often 3 |  |

**EXPOSURE TO COMMUNITY INTERVENTIONS**

| 701 | Have you heard of any community groups in this community who are helping in the area of the health of women and children? | No 0  Yes 1 | 707 |
| --- | --- | --- | --- |
| 702 | Have you participated in any community activity related to the health of women or children? | No 0  Yes 1 |  |
| 703 | Which community activity? | (specify) __________________________ |  |
| 704 | Please give me three examples of  messages related to the health of women or children that you learned about through these activities?  DO NOT READ OUT OPTIONS  SELECT ALL OPTIONS MENTIONED | *Add categories* |  |
| 705 | Did you later discuss what your experience with anyone? | No 0  Yes 1 | 707 |
| 706m | Who did you discuss your experience with?  DO NOT READ OUT OPTIONS  SELECT ALL OPTIONS MENTIONED | Wife A  Mother B  Male relative C  Friend D  Co-worker E  Religious or community leader F  Other (specify) _________________ X |  |
| 707m | A community activity that is being implemented in several communities in this area is the male mentor programme, men are trained to be mentors to other men, and talk with them about issues related to the health of women and children.  Have you ever heard about the male mentor activity? | No 0  Yes 1 | 712 |
| 708m | Have you ever been instructed by the male mentor, or do you know someone who was instructed? | No A  Respondent was mentored B  Respondent knows someone who was  mentored C |  |
| 709m | Do you think that male mentors can help men better understand the health issues of women and children? | No 0  Yes 1  Don’t know 98 |  |
| 710m | In your opinion, do male mentors have a positive or negative impact on the community? | Positive 1  Negative 2  Neither positive nor negative 3 |  |
| 711m | Do you think that male mentors can be beneficial for men in this community? | No 0  Yes 1  Don’t know 98 |  |
| 712m | If you were given the opportunity, will you want to be instructed by a male mentor? | No 0  Yes 1 |  |
| 713m | Another community activity that is being implemented in several communities in this area is the safe space group for young married women, which provides them with information about their health and the health of their children. Have you ever heard about the safe space group activity? | No 0  Yes 1 |  |
| 714m | Do you know a young woman who have participated in the safe space group activity? | No 0  Yes 1 |  |
| 715m | We talked a lot today about various issues related to the health of women and children. In the past year, where did you get information about maternal and child health?  DO NOT READ OUT OPTIONS  SELECT ALL OPTIONS MENTIONED | Did not get information in the past year 0  Male mentor 1  Religious leader 2  Health facility 3  TBA 4  Wife 5  Relatives 6  Friends 7  Other (specify) ____________________ 99 |  |

THANK PARTICIPANT FOR HIS TIME AND END INTERVIEW
