# Supplementary material for: Factors associated with knowledge of obstetric danger signs and perceptions of the need for obstetric care among married men in northern Nigeria: a cross-sectional survey
Source: BMC Pregnancy Childbirth. 2019 Apr 11;19:123. doi: 10.1186/s12884-019-2271-1 (PMC6458632; doi:10.1186/s12884-019-2271-1)
Supplement: Supplementary file 2 — Variables and computation. (DOCX 16 kb) [file 12884_2019_2271_MOESM2_ESM.docx]

**Appendix 1: Computation of variables used in analysis**

The following variables, including the details of their computation, were used for analysis in this study.

1. *Knowledge of danger signs in pregnancy.* We asked respondents what are some serious problems that can occur during pregnancy and probed to obtain a full list of danger signs the respondent knew of. Responses were coded into the following danger signs: bleeding, severe headache, blurred vision, convulsion, swollen hands or face, high fever, loss of consciousness, difficulty breathing, severe weakness, severe abdominal pain, accelerated or reduced foetal movement, and water breaks without labour. Our knowledge variable is coded 1 if the respondent listed two or more of these danger signs, 0 if he only mentioned one or none.
2. *Knowledge of danger signs in delivery*. We asked respondents what are some serious problems that can occur during labour and childbirth and probed to obtain a full list of danger signs the respondent knew of. Responses were coded into the following danger signs: severe bleeding, severe headache, convulsions, high fever, loss of consciousness, labour lasting more than 12 hours, and placenta not delivered soon after birth. Our knowledge variable is coded 1 if the respondent listed two or more of the danger signs, 0 if he only mentioned one or none.
3. *Knowledge of danger signs in the first two days postpartum.* We asked respondents what are some serious health problems that women can experience in the first two days after giving birth and probed to obtain a full list of danger signs the respondent knew of. Responses were coded into the following danger signs: severe bleeding, severe headache, blurred vision, convulsions, swollen hand or face, high fever, vaginal secretions that smell bad, loss of consciousness, difficulty breathing, and severe weakness. This variable is also coded 1 if the respondent listed two or more of the danger signs, 0 if he only mentioned one or none.
4. *Perception that antenatal care can reduce the risk of complications.* We asked respondents what are some things that a woman can do to reduce the risk of complications in pregnancy and childbirth. This variable is coded 1 if the responded mentioned antenatal care, 0 otherwise.
5. *Perception that women should deliver in a health facility or hospital.*  We asked respondents where, in their opinion, is the best place for women to deliver their babies. Our dependent variable is coded 1 if they answered: ‘in a health facility or hospital’, 0 otherwise.

The literature suggests a number of factors that can influence, or enable, better health knowledge and perceptions []. We operationalize these factors using the following variables:

*Age*. This is a continuous variable, ranging 18-80.

*Literacy* is used to measure education. This is a dichotomous variable, coded 1 if the respondent could read a simple sentence he was shown, 0 otherwise.

*Polygamy*. This is a dichotomous variable, coded 1 if the respondent had more than one wife, 0 otherwise. Respondents had between one and four wives, such that at least one was younger than 25 years.

*Number of children*. This is a continuous variable, ranging 0-38. It includes living children from all the man’s wives combined.

*Household wealth*. We created an index of household wealth, which was constructed by collecting information about whether respondent’s household owned, at the time of the survey, each of the following six items: wall clock, mobile phone, fan, radio, television, and refrigerator. We estimated the cost range in the study states at the time of the survey for each of these items. For example, the cost of the cheapest, used black-and-white television, and the cost of the most expensive new colour television. We then calculated a relative price index for each item, by: (1) dividing the lowest cost of the item by the lowest cost of the cheapest item (wall clock), (2) dividing the highest cost of the item by the highest cost of the cheapest item, and (3) averaging the two. Finally, we used the following formula to calculate the value of the wealth index for each respondent.

$${Index}_{i}= \sum_{i} {OWN}_{ij}\left( \frac{{RPI}_{j}}{\sum_{j} {RPI}_{j}} \right)$$

Where:

OWN_ij_ is a dichotomous variable taking the value of 1 if household owned the good j, and zero otherwise;

RPI_j_ is the relative price index of good j.

This resulted in an index ranging 0 – 100, where 100 means the household owned all five items, with a mean of 28.5 and median of 14.7. Because the distribution of this continuous variable is very far from normal, as indicated by the difference between the mean and median, and how far they both are form 50, we constructed a series of dummy variables, based on index quartile, to control for household wealth in the multivariate analysis. The base category is the lowest quartile.

*Electricity*. This is another measure of household wealth, coded 1 if the respondent’s home was connected to electricity, 0 otherwise.

Wife *experience of complications in: (1) pregnancy, (2) labour and delivery, and (3) the first two days postpartum.* Each of these three variables was coded 1 if the respondent said that any of his wives had ever experienced any of the complications included in the corresponding knowledge variable, 0 otherwise.

*State*. This variable controls for the state the respondent lives in, coded 1 if Kaduna, 0 if Katsina.
